# Cosolute effects reveal the nature of weak forces governing GLP-1 oligomer stability

**DOI:** 10.1016/j.jbc.2026.111223

**Published:** 2026-02-02

**Authors:** Anyah Settle, Rahul Mishra, Ramesh Kumar Shanmugam, Viv Lindo, Nathan BP. Adams, Thomas A. Jowitt, Tuck Seng Wong, Barbara Ciani

**Affiliations:** 1 Centre for Chemical Biology, School of Mathematical and Physical Science, University of Sheffield, Sheffield, UK; 2 BioPharmaceuticals Development, Analytical Sciences, AstraZeneca, Cambridge, UK; 3 Biopharmaceutical Development, Dosage Form Design and Development, AstraZeneca, Cambridge, UK; 4 NanoTemper Technologies GmbH, Munich, Germany; 5 Wellcome Centre for Cell-Matrix Research, Faculty of Biology, Medicine and Health, Manchester Academic Health Science Centre, University of Manchester, Manchester, UK; 6 School of Chemical, Materials and Biological Engineering, University of Sheffield, Sheffield, UK

**Keywords:** GLP-1, protein aggregation, colloidal stability, physical stability, amyloid, self-assembly

## Abstract

Glucagon-like peptide-1 (GLP-1) is an incretin hormone widely used to manage diabetes and obesity through its ability to regulate glucose homeostasis. Clinically relevant GLP-1 sequences form oligomeric states. Uncontrolled oligomer formation can drive fibril formation, posing challenges, such as difficulty in controlling drug dosage, loss of activity, or toxicity, as the aggregates can be immunogenic and/or can form amyloids. Here, we used combined measurements of colloidal and conformational stability to characterize the intermolecular interactions underpinning the physical status of the GLP-1(7–37) amide (GLP-1am), at pharmaceutically relevant high concentrations. We focus on less explored conditions, around pH 5, mimicking the environment within native cellular secretory granules, where the hormone is also densely packed. Cosolutes allowed us to interfere with weak interactions affecting peptide self-association into soluble oligomers and the conversion into aggregates and fibrils. We show that GLP-1am exists as soluble oligomers that assemble into nanosheets over the timescale of hours, in quiescent conditions. Aggregation proceeded *via* a nucleation-dependent mechanism, with its rate correlating to the magnitude of attractive intermolecular interactions. It was accelerated by ionic cosolutes, indicating a key role for screening of electrostatic interactions in modulating peptide–peptide attraction and assembly. The rate of aggregation was also pH dependent, with rates being slower at pH 5 than pH 8. Notably, the addition of proline, as a cosolute, delayed the onset of GLP-1am aggregation in a pH-dependent manner. Thus, in quiescent conditions, GLP-1am forms discrete soluble oligomers capable of organizing into ordered nanostructures rather than amyloid fibrils.

Glucagon-like peptide-1 (GLP-1) is an incretin hormone that plays a crucial role in regulating glucose homeostasis. The GLP-1 peptide binds to the glucagon receptor, a member of the B family of seven transmembrane G-protein–coupled receptors (1). This receptor is an effective target for lowering elevated blood glucose levels associated with type 2 diabetes (2). GLP-1 receptor agonists (GLP-1RAs) stimulate insulin secretion, suppress glucagon release, and delay gastric emptying in a glucose-dependent manner (3). However, the native peptide sequences have a short circulating lifetime (*t*_1/2_ ∼2 min after intravenous administration) because of cleavage of the peptide’s N terminus by dipeptidyl peptidase IV, resulting in deactivation (2). Strategies for improving the half-life and uptake of GLP-1RAs include the addition of lipids and sequence modifications, such as substitution with noncanonical amino acids at protease cleavage sites. In the case of liraglutide, a common GLP-1RA antidiabetic and obesity medication, adding a lipid tail increases the half-life to 9 to 13 h (4).

GLP-1RAs have demonstrated broader benefits, such as significantly reducing the risk of heart failure, atherosclerosis, and hypertension (5). Consequently, there is a broad interest in providing agonists, which are more stable and effective under physiological conditions both prior to and following administration.

GLP-1 peptides have a tendency to self-associate into reversible oligomers, leading to delayed absorption into systemic circulation, where monomers bind to albumin (6, 7). Oligomerization improves stability and activity (8, 9) when maintained in circulation. Similarly, GLP-1’s biological activity is regulated by the slow release of monomers from hormone fibrils stored in the secretory granules (10). Due to the propensity for self-association, GLP-1RAs are challenging to formulate in conditions that retain physical stability during storage (11) and avoid aggregated states that could compromise therapeutic efficacy (12).

Since the discovery of GLP-1’s therapeutic potential, the mechanisms of aggregation (13, 14, 15, 16) have been explored (11, 14). However, a gap in understanding remains regarding which intermolecular interactions underpin GLP-1 oligomerization and how cosolutes can influence its aggregation propensity. This knowledge is crucial for designing strategies to either develop next-generation GLP-1RAs and/or select solution conditions that control oligomeric species and inhibit aggregation, thus preventing the formation of immunogenic species.

The initial step in the kinetics of protein self-association leading to either amorphous aggregation or organized fibril formation involves the formation of soluble oligomers. The pathway taken depends on the solution conditions experienced by the polypeptide chain, which can favor β-amyloid (Aβ) structures (17, 18).

GLP-1(7–37), GLP-1(7–36) amide, and GLP-1(7–37) amide (GLP-1am) show similar receptor affinity and activation but slightly different pathways to amyloid fibrillation (19, 20), which have been studied around physiological pH and at low peptide concentrations (21, 22). GLP-1am self-associates at low concentrations (0.3 mg mL^–1^) upon mechanical agitation, forming distinct oligomers containing three to four peptide subunits (22). These oligomers appear thermally stable and are not impacted by sonication but denature in the presence of anionic surfactants, whereas nonionic surfactants drive oligomerization further. However, most commercial human GLP-1RA formulations consist of highly concentrated solutions (4) (1.3–6 mg mL^–1^) at mildly alkaline pH, whereas native GLP-1 is stored in the crowded environment of cellular secretory granules at mildly acidic pH. This ensures both slow release and controlled activity. Importantly, physical stability studies of GLP-1 in formulation-relevant and natural storage conditions are lacking, and this knowledge could provide the basis for precise control of GLP-1 self-association and monomer release from its assembled state.

The physical behavior of a protein in solution is determined by its colloidal and conformational stabilities, both of which are regulated by intermolecular and intramolecular noncovalent interactions (17). Dynamic light scattering (DLS) and determination of the diffusion interaction parameter (*k*_*D*_) (23) are methods of choice to quantify colloidal stability. The *k*_*D*_ directly correlates with protein diffusivity in solution, which, in turn, depends on the magnitude of protein–protein interactions (24). Nano differential scanning fluorimetry (nanoDSF) allows the measurement of conformational stability by following protein unfolding induced by temperature *via* intrinsic fluorescence.

Herein, we report the use of high-throughput combined light scattering (DLS and turbidity) and nanoDSF measurements to characterize the physical stability of GLP-1am at concentrations relevant to therapeutic formulations, at secretory granule-relevant pH (pH 5.5–6), under quiescent conditions (25).

We unravel the intermolecular interactions driving peptide oligomerization using selected, pharmaceutically relevant cosolutes and characterize how biologically relevant storage conditions control the morphology and the kinetics of monomer self-assembly to form GLP-1am fibrils.

We show that in the absence of mechanical stress, GLP-1am forms large nanostructures as opposed to fibrils. Sodium chloride (NaCl) drives steady aggregation of GLP-1am, whereas the salt form of arginine (arginine.HCl) drives self-association at high cosolute concentrations. Significantly, proline appears to slow down the growth of aggregates by stabilizing early oligomeric species. Thus, we present how the impact of cosolutes on GLP-1am self-assembly gives indications on the nature of the intermolecular interactions responsible for the early stages of self-association.

## Results

### Attractive interactions govern GLP-1am oligomer growth in a pH-dependent manner

**Figure 1:**
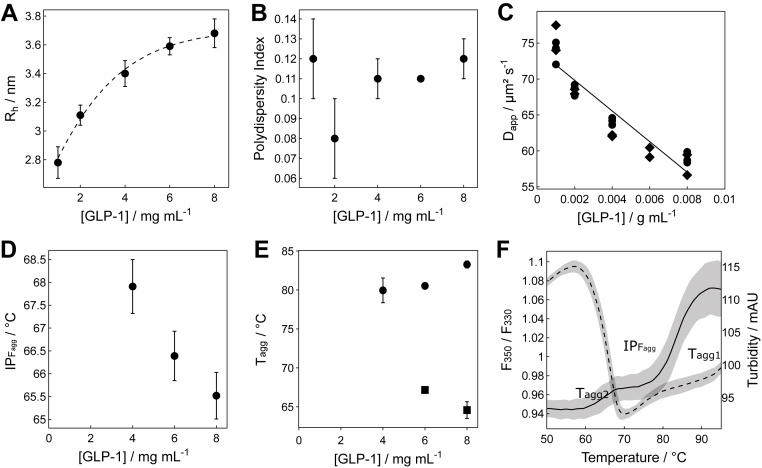
**Self-association of GLP-1am in 25 mM sodium acetate, pH 5**. *A*, apparent hydrodynamic radius, *R*_h_, of peak 1 (see Fig. S1*E* for peak labels) and (*B*) corresponding polydispersity index (PDI), of GLP-1am in 25 mM acetate buffer pH 5 and 20 °C as a function of its concentration measured with the Prometheus Panta (NanoTemper). The PDI is always lower than the 0.2 limit for a monodisperse sample. *C*, diffusion coefficient of the main GLP-1am species (peak 1) as a function of GLP-1am concentration. A total of five capillaries per concentration (except for 6 mg mL^-1^) were repeated over 2 days. For each capillary, the average of 10 acquisitions was displayed as a data point. *D*, inflection points of the 350 nm to 330 nm intrinsic fluorescence emission ratio (IPF_agg_) of GLP-1am as a function of peptide concentration. No inflection point (IP) could be seen below 4 mg mL^-1^; see Fig. S3*A* for raw traces. *E*, two IPs mark the appearance of turbidity during thermal ramp (1 °C min^-1^) in GLP-1am samples from 4 mg mL^-1^. The IP at lower temperature (T_agg2_, *squares*) decreases with increasing peptide concentration, whereas the IP at higher temperature (T_agg1_, *circles*) increases. See the corresponding raw data in Fig. S3*C*. *F*, the IP temperature (IP_Fagg_) for the ratio between the intrinsic fluorescence at 350 nm and 330 nm (*dashed line*) corresponds to the first increase in turbidity for GLP-1am 8 mg mL^-1^. Average and standard deviation of three repeats are shown. GLP-1am, GLP-1(7–37) amide.

We employed simultaneous measurements of size distribution, thermal unfolding, fluorescence, scattering, and turbidity to quantify the colloidal and conformational stability of GLP-1am in acetate buffer at pH 5, at concentrations from 1 to 8 mg mL^–1^. The size distribution profile of GLP-1am dilutions from a high concentration stock showed the presence of several oligomeric species (Fig. S1), some persistent, and some present only at the highest concentration (100 nm > *R*_h_ < 1000 nm; Fig. S1*E*). The hydrodynamic radius (*R*_h_) of the main GLP-1am species (Fig. S1, peak 1) increased with increasing peptide concentration, from 2.8 to 3.8 nm between 1 and 8 mg mL^–1^ (Fig. 1*A*). The estimated *R*_h_ of GLP-1am (∼2.15 nm), calculated from its hydrodynamic volume using the convex method (26) (Protein Data Bank code: 3IOL) suggested that the main species (peak 1) likely represents a combination of monomers and oligomers with sizes too similar to be resolved by DLS. Although high-throughput measurements indicated that the main population (peak 1) of GLP-1am is monodisperse (Fig. 1*B*), detection at a different scattering angle (Fig. S2, *A* and *B*) confirmed GLP-1am was polydisperse (Table S1). The concentration-dependent data allowed us to calculate the diffusion interaction parameter (*k*_*D*_) to quantify the interactions between GLP-1am molecules in the population with *R*_h_ in the range of 2.8 to 3.8 nm (Fig. 1*C*; *k*_*D*_ value around −29 ± 4 mL g^–1^). The sign and magnitude of the *k*_*D*_ value determine the intermolecular interactions: negative values indicate attraction, whereas positive values indicate repulsion (27, 28). For GLP-1am in acetate buffer at pH 5, the *k*_*D*_ was negative, a finding consistent across high- and low-throughput measurements (Fig. S2*C*, *k*_*D*_ value around −32 ± 9 mL g^–1^). This confirmed the presence of attractive interactions at pH 5, albeit weaker than those observed at pH 8 (Fig. S2, *D*–*F*; *k*_*D*_ = −42 ± 8 mL g^–1^).

To quantify the thermal stability of oligomeric species, we measured the change of intrinsic fluorescence of aromatic residues with thermal stress. From 20 to ∼60 °C, GLP-1am showed a linear increase in the ratio of fluorescence intensity at 350 nm to 330 nm (*F*_350_/*F*_330_), indicating unfolding of weakly associated peptide oligomers (Fig. S3*A*). At peptide concentrations of 4 mg mL^–1^ and above, we observed a sharp decrease in the *F*_350_/*F*_330_ with a simultaneous increase in scattering intensity (Fig. S3*B*) and of turbidity (Fig. S3*C*) from ∼55 °C (Fig. S3, *A* and *D*). These events suggested a burying of the aromatic residues due to the formation of large species, which occurred at increasingly lower temperatures with increasing peptide concentration (Fig. 1, *D* and *E*). The temperature marking this assembly event (IP_Fagg_) coincided with the first appearance of turbidity (Fig. 1*F*; Fig. S3*D*), reflecting the formation of particles >12.5 nm. The turbidity signal showed two distinct transitions (T_agg1_ and T_agg2_), indicating the growth of oligomers, with larger species being more thermally stable at higher concentrations (T_agg1_, Fig. 1, *E* and *F* and Fig. S3*C*). This event coincided with the appearance of a second species in the size distribution during the thermal ramp, just as the smaller species disappeared around ∼57 °C. This new species, around 100 nm *R*_h_, became more populated until it also disappeared at ∼76 °C (Fig. S3*E*), at which point the intensity of scattered light became too great to measure using DLS.

We utilized low-throughput fluorescence measurements to further probe the environments of the aromatic residues in GLP-1am. The emission of the freshly dissolved GLP-1am at 340 nm (Fig. S3*F*) suggested that the aromatic residues were partially buried in a hydrophobic environment, confirming the presence of oligomers (29). After days of incubation without agitation, at 25 °C, GLP-1am aromatic residues displayed a decrease in emission intensity at both pH 5 and pH 8 (Fig. S3, *F* and *G*) with a small blue shift of 2 nm in emission, highlighting further changes to GLP-1am oligomer conformation.

Last, we sought to define how solution conditions impact the self-association behavior of GLP-1am by exploiting electrophoretic, spectroscopic, and chromatographic methods across a range of pH conditions. The ζ-potential, which describes the surface charge, was near zero for GLP-1am at approximately pH 6.2 (Fig. S4*A*), close to the previously measured isoelectric point of 6.8 (22). The surface charge followed the curve of the net charge between pH 3.7 and 6 but then declined rapidly between 6 and 8. Overall, the ζ-potential values for GLP-1am fell within the ±30 mV range, indicating poor colloidal stability and a general tendency to self-association between pH 3 and 8. This was consistent with GLP-1am being larger than expected for a monomer at any pH (Fig. S4*B*), with the smallest *R*_h_ measured at pH 4 and the largest at pH 7. The polydispersity was invariant with respect to pH, except at pH 8, when it decreased below 16% (Fig. S4*C*), but with a large variation in hydrodynamic size. Asymmetric flow field flow fractionation with multi-angle light scattering (AF4–MALS) reported GLP-1am as a large oligomer between 1 and 4 mg mL^-1^ (Table S2). Consistent with the hydrodynamic measurements, far-UVCD showed that GLP-1am helical content at pH 4 is lower than that at pH 5 and 8 (Fig. S4*D*). Size-exclusion chromatography (SEC) was used to estimate GLP-1am existed as a dimer at pH 8 at 0.3 mg mL^-1^ (Fig. S4*E*, Table S4).

Thus, GLP-1am shows poor colloidal stability across a wide pH range, providing a driving force for self-association. This assembly process is concentration dependent, resulting in differential thermal stabilities where small oligomers become less stable with concentration, whereas larger aggregates become more stable. GLP-1am oligomer size and conformation are also pH dependent, with attractive interactions driving self-association being weaker at pH 5 than pH 8.

### The impact of salt and osmolytes on the physical stability of GLP-1am

To probe the nature of the intermolecular interactions that drive GLP-1am self-association, we examined the effect of cosolutes on the physical stability of GLP-1am at pH 5. NaCl was chosen to disrupt electrostatic interactions, whereas arginine.HCl and proline were employed to interfere with the formation of oligomers driven by a combination of electrostatics, cation−π, and hydrophobic interactions (30).

**Figure 2:**
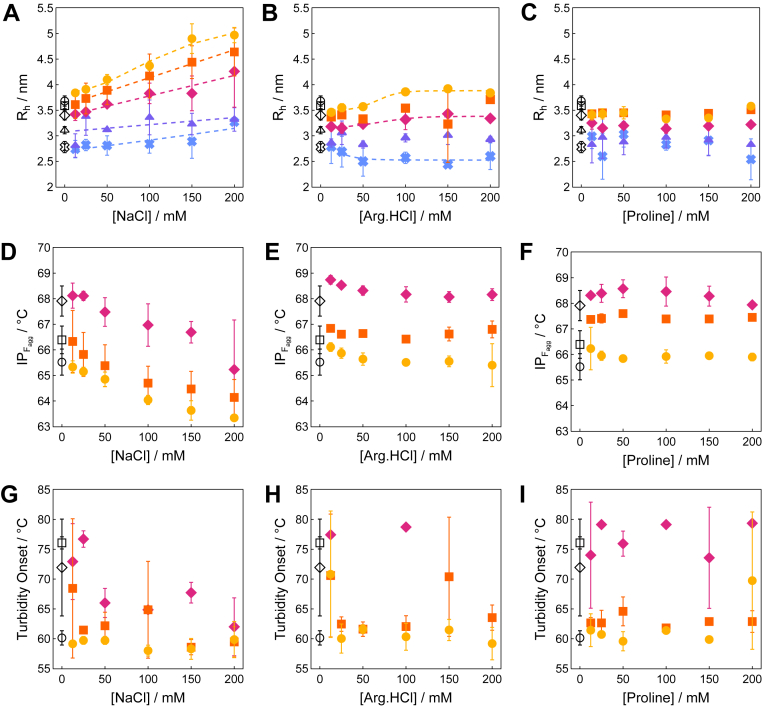
**High-throughput screening of effect of cosolutes on the self-association of GLP-1am at pH 5.** Apparent hydrodynamic radius of GLP-1am as a function of peptide concentration in the presence of (*A*) sodium chloride (NaCl), (B) arginine.HCl, and (*C*) proline. A Welch's *t* test, comparing pooled proline and acetate data for concentrations ≥2 mg mL^-1^ found a significant effect of the cosolute (Welch’s *t*(14.4) = 2.59, *p* = 0.011, Cohen’s *d* = 0.822). Inflection points for the 350 nm to 330 nm ratio of the intrinsic fluorescence emission of GLP-1am as a function of peptide concentration in (*D*) NaCl, (*E*) arginine.HCl, and (*F*) proline. Onset temperature for the turbidity of GLP-1am as a function of peptide concentration in (*G*) NaCl, (*H*) arginine.HCl, and (*I*) proline. All samples in 25 mM acetate buffer pH 5. Average and standard deviation of three repeats are shown. Peptide concentration: 8 mg mL^-1^ (*circle*, *yellow*), 6 mg mL^-1^ (*square*, *orange*), 4 mg mL^-1^ (*diamond*, *pink*) 2 mg mL^-1^ (*triangle*, *purple*), and 1 mg mL^-1^ (*cross*, *blue*). Samples prepared in acetate buffer alone are shown in *black*. GLP-1am, GLP-1(7–37) amide.

Characterization of the colloidal status of GLP-1am by DLS showed a varying effect on its hydrodynamic size depending on the cosolute. Increasing the NaCl concentration led to a large increase in the *R*_h_ of GLP-1am. The effect of NaCl was also greater at higher concentrations of GLP-1am, whose size increased almost linearly with salt concentration (Fig. 2*A*). In contrast, the impact of arginine.HCl was highly dependent on peptide and cosolute concentration (Fig. 2*B*). The *R*_h_ of GLP-1am decreased up to 50 mM arginine.HCl and GLP-1am concentrations lower than 4 mg mL^–1^. Conversely, the *R*_h_ increased slightly up to 100 mM cosolute above 4 mg mL^–1^ GLP-1am but remained smaller than that for acetate alone. The similarity between the impact of NaCl and arginine.HCl on GLP-1am size is likely due to their similar charge-screening ability when they are at sufficiently high concentrations. In contrast, at low concentrations, only arginine.HCl can provide some colloidal stabilization, possibly by preferentially interacting with aromatic side chains, a similar mechanism observed in larger proteins (31).

Proline cannot interfere with ionic interactions; thus, we hypothesized that proline would only have an impact if hydrophobic interactions play a role in GLP-1am self-association. The effect of proline on GLP-1am size appeared negligible in comparison to the effect of ionic cosolutes (Fig. 2*C*). However, we observed a pooled effect of proline that led to a modest but statistically significant reduction in *R*_h_, across most GLP-1am concentrations (mean *R*_h_ 3.22 nm *versus* mean *R*_h_ 3.43 nm for GLP-1am in proline and acetate, respectively; standard errors are 0.03 nm and 0.07 nm for measured *R*_h_ in proline and acetate, respectively). At peptide concentrations of 2 mg mL^–1^ and above, there was a consistent decrease in GLP-1am hydrodynamic radius in the presence of proline in comparison to that in acetate buffer alone. This effect, though small in magnitude, was consistent across conditions; thus, proline seems to favor assemblies with a smaller average size between 2 and 8 mg mL^–1^ GLP-1am.

Characterization of the thermal stability of GLP-1am with nanoDSF did not show any significant effect of the cosolutes on peptide unfolding profiles (Fig. S5). However, the effect of cosolutes on the midpoint temperature of aggregation, IP_Fagg_, was clear. The decline in IP_Fagg_, accompanied by the simultaneous increase in turbidity, and peptide size increased with increasing NaCl concentration (Fig. 2*D*, Fig. S5, *A*–*E*). Thus, NaCl promoted the formation of large aggregated species at lower temperatures than in buffer alone. In the presence of arginine.HCl, the GLP-1am IP_Fagg_ showed a small increase at 12.5 mM, followed by a subsequent decrease that plateaued at 50 mM (Fig. 2*E*, Fig. S5, *F*–*L*). In comparison to NaCl, arginine.HCl displayed a shallower decrease of the IP_Fagg_ (1 °C for arginine.HCl *versus* 2 °C for NaCl, compare Fig. 2, *D* with *E*). Similarly to arginine.HCl, IP_Fagg_ increased up to 100 mM proline concentration, suggesting that proline reduces the tendency of GLP-1am to self-associate with temperature. This effect was less obvious at 8 mg mL^–1^ GLP-1am, where, perhaps, interactions between GLP-1am molecules were predominant (Fig. 2*F*, Fig. S5, *J*–*O*). It was also apparent that the higher the peptide concentration, the lower the turbidity onset temperature, which is typical of nucleation-dependent mechanisms (Fig. 2, *G*–*I*, Fig. S5*G*).

**Figure 3:**
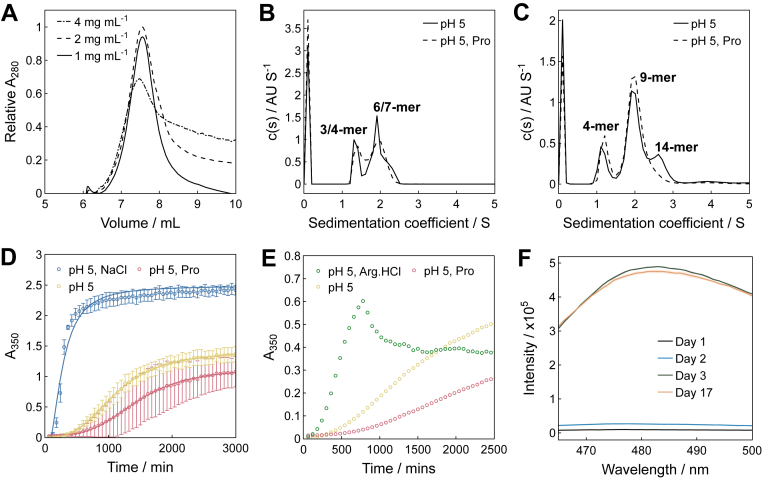
**Concentration-dependent oligomerization and aggregation kinetics of GLP-1am.***A*, AF4–MALS analysis of a 1 to 4 mg mL^-1^ GLP-1am fresh samples in 25 mM acetate buffer (pH 5). The molecular weight determination can be found in Table S2. *B*, sedimentation velocity analytical ultracentrifugation (SV–AUC) of GLP-1am 2 mg mL^-1^ without and with 100 mM proline, in 25 mM acetate buffer at pH 5. *C*, SV–AUC of GLP-1am 4 mg mL^-1^ in 25 mM acetate buffer at pH 5, without and with 100 mM proline. SV–AUC analysis in Table S3. *D* and *E*, aggregation kinetics of 2 mg mL^-1^ GLP-1am in 25 mM acetate buffer (pH 5) without and with cosolutes monitored by apparent absorbance at 350 nm, at 25 °C, in quiescent conditions. Average and standard deviation of two technical repeats shown in *D*. *F*, aggregation kinetics of 1 mg mL^-1^ GLP-1am monitored by thioflavin T binding, at 25 °C, with agitation. AF4–MALS, asymmetric flow field flow fractionation–multiangle light scattering; GLP-1am, GLP-1(7–37) amide.

Subsequently, we sought to understand how cosolutes corresponded to the peptide's propensity to form high–molecular weight species. AF4–MALS and SEC indicated that GLP-1am at pH 5 contained dimeric and trimeric species, up to 2 mg mL^-1^, alongside aggregates that could not be recovered (Fig. 3*A*, Tables S2, S4). Sedimentation velocity analytical ultracentrifugation (SV–AUC) was used to characterize the effect of proline on the formation of large GLP-1am species. SV–AUC showed that whilst proline had a small effect on GLP-1am oligomer formation at 2 mg mL^-1^ peptide (Fig. 3*B*, Table S3), it could prevent the formation of the largest aggregates at a higher peptide concentration. When proline was added to 4 mg mL^–1^ GLP-1am, the number of oligomeric species reduced from three (4-, 9-, and 14-mers) to two, with the loss of the 14-mer population (Fig. 3*C*; Table S3). To enable direct comparison and to observe the true effect of proline, each 4 mg mL^–1^ GLP-1am sample was prepared from the same stock solution with and without proline. Thus, the loss of the 14-mer demonstrates unequivocally that the reduction reflects a true effect, suggesting that proline likely slowed down the self-association of GLP-1am.

Oligomer growth kinetics, monitored by apparent absorbance at 350 nm, at 25 °C, showed that the size of GLP-1am increased faster when arginine.HCl and NaCl were included in the buffer (Fig. 3, *D* and *E*). In contrast to ionic cosolutes, GLP-1am had a slightly delayed onset of turbidity in the presence of proline, which plateaued at lower absorbances (Fig. 3, *D* and *E*). Kinetic measurements were performed using batches purchased from GL Biochem (batch 1) and GenScript (batch 2). The effects of cosolutes on the rate of peptide aggregation were reproducible across peptide preparations (compare Fig. 3, *D* and *E* with Fig. S7, *A*–*C*), even though absolute rates were batch dependent. Adding small amounts of seeds (Fig. S7, *B* and *C*) or increasing peptide concentration (Fig. S7, *D* and compare *A* with *E*) accelerated the aggregation of GLP-1am in both batches, consistent with a nucleation-dependent self-assembly pathway. Albeit to a lesser extent than its salt form, zwitterionic arginine also accelerated GLP-1am aggregation (Fig. S7*E*). The kinetics were found to be faster at pH 8 than at pH 5, reflecting the greater magnitude of the previously discussed *k*_*D*_ value and, thus, stronger attractive interactions. Oligomer growth was also faster at pH 8 in comparison to pH 5, reflecting the stronger attractive interactions as quantified by the *k*_*D*_. Notably, the effect of proline was pH dependent since the addition of proline to GLP-1am at pH 8 had no impact on the aggregation rate (Fig. S7*F*). Overall, the growth kinetics were repeatable over different days within the same batch (Fig. S7*G*). Oligomer growth kinetics were supported by SV–AUC of 4 mg mL^–1^ GLP-1am assembled over 18 h, at 25 °C, which showed that the higher molecular weight species were slightly smaller when proline was present (Fig. S7*H* and Table S3). Importantly, GLP-1am formed amyloid-like fibrils only when incubated for days with agitation (Fig. S7*I*).

**Figure 4:**
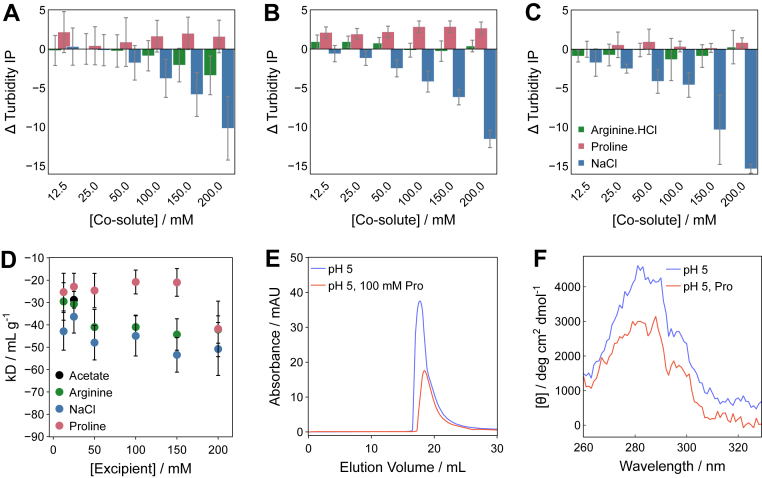
**Effect of cosolutes on the colloidal and conformational Stability of GLP-1am.** Difference in inflection point (IP) temperature for the appearance of the major turbidity transition (T_agg1_) for GLP-1am samples with excipient to those in 25 mM acetate buffer only at pH 5 at (*A*) 4 mg mL^-1^, (*B*) 6 mg mL^-1^, and (*C*) 8 mg mL^-1^.A positive difference indicates a higher temperature for aggregate formation in the presence of excipient, hence, a lower propensity to aggregate. *D*, diffusion interaction parameter, *k*_*D*_, for GLP-1am in the formulation with 25 mM acetate buffer at pH 5 and excipients. The data displayed are from the linear regression of individual diffusion coefficients in varying cosolute concentrations, performed as indicated in the Experimental procedures section. Average and standard deviation of three repeats are shown. *E*, SEC of GLP-1am prepared without or with 100 mM proline. The samples were separated with a Superdex 75 10/300 GL at 0.7 mL min^-1^ in 25 mM acetate buffer (pH 5) mobile phase, without or with 100 mM proline as appropriate (0.5 ml of 0.3 mg mL^-1^ injection). Molecular weight estimations can be found in Table S4. *F*, near-UV CD of 2 mg mL^-1^ GLP-1am in 25 mM acetate buffer in the absence or presence of 100 mM proline. GLP-1am, GLP-1(7–37) amide; SEC, size-exclusion chromatography.

The T_agg1_ trends measured upon changing temperature, with cosolutes, recapitulated the general propensity of GLP-1am to oligomerize at constant temperature. Whilst arginine.HCl generally decreased T_agg1_, proline raised this value across concentrations (Fig. 4, *A* and *B*). Ultimately, none of the cosolutes raised the T_agg1_ for the highest GLP-1am concentration (Fig. 4*C*). Interestingly, ionic cosolutes displayed only one turbidity transition upon changing temperature, rather than the two observed with proline or buffer alone (Fig. S6). The T_agg1_ trends also reflected the ability of each cosolute to decrease or increase the *k*_*D*_ for GLP-1am, with proline being the most effective in reducing attractive interactions (Fig. 4*D*). To rationalize the effect of proline on GLP-1am oligomer growth into aggregates, we looked at oligomer size and conformation in the presence and absence of proline. At low peptide concentration, SEC identified the dimer–trimer tipping point around 2 mg mL^–1^ peptide (Table S4). Proline appeared to favor GLP-1am dimers, compared with the trimers observed in buffer alone (Fig. 4*E*, Table S4). We then used CD to quantify the conformation of GLP-1am oligomers with and without proline. Near-UV CD absorption revealed that fresh GLP-1am adopts a distinct tertiary structure that constrains aromatic residues (Fig. 4*F*). The near-UV CD signal at 290 nm was reduced in the presence of proline, suggesting this cosolute promotes a change of environment for aromatic side chains. Taken together, the SEC and CD data suggest that proline favors smaller GLP-1am sizes at pH 5.

**Figure 5:**
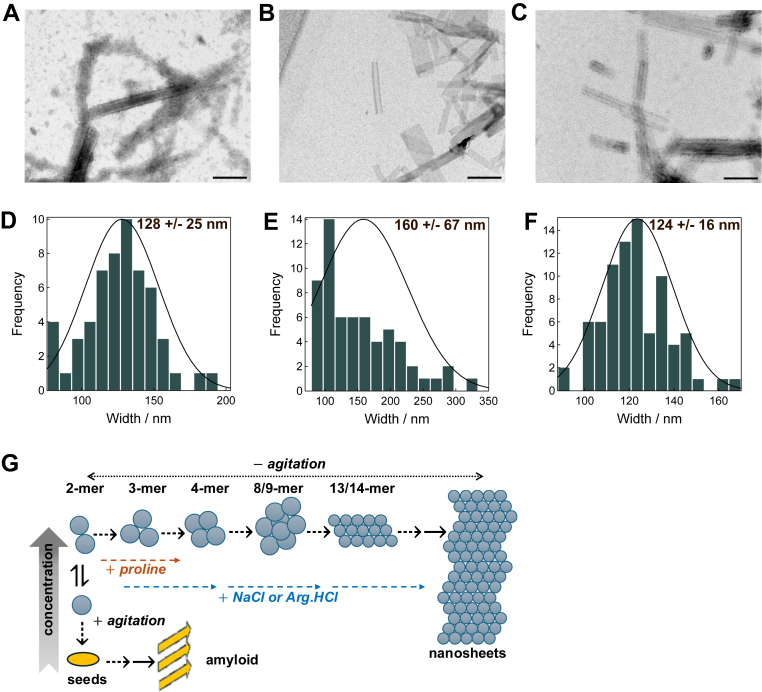
**Cosolutes affect the morphology of aggregates of GLP-1am at pH 5.** Transmission electron microscopy and width binned distribution of the nanosheets formed from 2 mg mL^-1^ GLP-1am incubated at 25 °C, without agitation, for 18 h in (*A*, *D*) 25 mM acetate buffer (pH 5) (n = 19); (*B*, *D*) 25 mM acetate buffer (pH 5) with 50 mM arginine.HCl (n = 21; and (*C*, *E*) 25 mM acetate buffer (pH 5) with 100 mM proline (n = 27). The scale bar represents 500 nm. Three measurements per nanosheet were taken (two at the edges and one in the middle of each structure measured). *G*, oligomerization pathway under quiescent and agitated conditions for GLP-1am at pH 5. Under quiescent conditions (–agitation, *top pathway*), GLP-1am exists as 2-mers and 3-mers (as detected by SEC and AF4–MALS). These species grow into 4-mers and then 8/9-mer and 13/14-mer species (characterized by SV–AUC), which ultimately associate into nanosheets. Ionic cosolutes (NaCl, arginine.HCl) accelerate nanosheet formation, implying electrostatic screening as a driver of aggregation. Conversely, the presence of proline delays growth into aggregates. Under agitated conditions (+ agitation, *bottom pathway*), GLP-1am forms protofibrils, which subsequently mature into amyloid structures (this work and (21)). The *arrows* indicate inferred transitions between experimentally observed states. GLP-1am, GLP-1(7–37) amide. AF4–MALS, asymmetric flow field flow fractionation–multiangle light scattering; GLP-1am, GLP-1(7–37) amide; SEC, size-exclusion chromatography.

Last, we examined the morphology of the GLP-1am aggregates formed after overnight incubation at 25 °C, under quiescent conditions. In contrast to the amyloid fibrils formed by GLP-1am at low concentration over days, with mechanical agitation (Fig. 3*F* and (22)), GLP-1am formed straight and flat nanosheets (Fig. 5*A*). These nanosheets appeared shorter and more geometrically defined when arginine.HCl (Fig. 5*B*) and proline were included in the buffer (Fig. 5*C*). At pH 8, the nanosheets spanned a broader distribution, exhibiting two populations with distinct widths (Fig. S8, *A* and *B*). The morphology of the mature nanosheets was identical across peptide batches and pH conditions (Fig. S8, *C* and *D*), suggesting that these structures are favored by either higher peptide concentration or the lack of mechanical stress during assembly. Analysis of the width distribution revealed that nanostructures formed at pH 5 (Fig. 5*D*) have a narrower distribution than those formed in arginine.HCl (Fig. 5*E*), whereas proline generated nanosheets more homogeneous in width (Fig. 5*F*). Interestingly, 200 mM proline promoted populations with two distinct width distributions: one with narrower nanosheets than those observed at 100 mM proline, and one distribution with widths similar to those promoted by arginine.HCl.

In summary, these results show that proline modulates the balance between oligomer formation and growth. GLP-1am oligomers formed in the presence of proline likely adopt a conformation less capable of growing into larger structures over time and form more homogeneous nanostructures than in buffer alone.

## Discussion

### GLP-1am self-assembles into organized nanosheets in quiescent conditions, with pH-dependent kinetics

We showed that GLP-1am self-associates into soluble oligomers at pH 5, with the oligomer size increasing with increasing peptide concentration. The kinetics of aggregation at 25 °C is concentration dependent, which is consistent with a nucleation-dependent mechanism, as reported previously (31). GLP-1am aggregation is accelerated at pH 8, with 8 mg mL^–1^ of GLP-1am aggregating within 1 hour. The aggregation propensity of GLP-1am is well predicted by the strength of the attractive intermolecular interactions, which are stronger for GLP-1am at pH 8 than pH 5. The nucleation-dependent kinetics at pH 8 agrees with what has been observed for the nonamidated peptide sequence (21), with mechanical agitation.

GLP-1am aggregation accelerates with seeding and concentration, confirming a nucleation-dependent mechanism. Under quiescent conditions and high peptide concentration, we did not observe the formation of twisted amyloid fibrils typical of cross-Aβ structures. A similar behavior was shown for Aβ sequences, where an increase in concentration led to a decrease in fibrillation propensity (32, 33). At high concentrations, GLP-1am forms straight nanosheets after days of incubation at room temperature, similar to what is observed for glucagon (34). The straight morphology of these peptide assemblies must depend on GLP-1am accessing oligomeric structures, leading to a different pathway than that of amyloid formation. This contrasts with amyloid formation, which instead requires a degree of unfolding to create seeds, which are normally generated by agitation. Thus, at high peptide concentrations, GLP-1am behaves like glucagon (34) and other reversible fibrillar aggregates formed by incretin hormones (10).

### The chemistry of the cosolutes reveals the nature of intermolecular interactions driving GLP-1am oligomerization

NaCl steadily increases GLP-1am aggregation propensity, with a corresponding decrease in the IP_Fagg_, which can be explained by the screening of several charged patches present on the electrostatic surface of GLP-1am at pH 5 (Fig. S9*A*). Direct relationships between NaCl concentration and acceleration of fibril formation have been observed before, especially for peptides (35).

Like NaCl, arginine.HCl can negatively impact protein solubility by screening repulsive intermolecular interactions, leading to the destabilization of proteins with high surface charge (36). We saw this effect at 50 mM arginine.HCl, where the rate of aggregation was increased. Where NaCl drove an increase in size for all GLP-1am concentrations, the effect is more complex for arginine.HCl. Below 4 mg mL^-1^ GLP-1am, and when the arginine.HCl exceeded 50 mM, GLP-1am size decreased (Fig. 2*B*). Thus, at high peptide concentrations, arginine.HCl primarily acts as a charge screen to reduce repulsion and promote self-association. At lower peptide concentrations, arginine.HCl might engage in preferential interactions with exposed aromatic, and to a lesser extent, with hydrophilic residues to reduce oligomer size (30). Depending on protein size and cosolute concentration (below or above 100 mM) (37), these interactions can stabilize or destabilize proteins. The dual effect of arginine.HCl on GLP-1am may arise from oligomers with partially buried aromatic side chains, which are more prevalent at higher peptide concentrations and permit electrostatic screening effects to dominate self-association behavior.

Taken together, our results indicate that NaCl promotes GLP-1am oligomer growth by electrostatic screening, producing the largest increase in size and aggregation propensity. By contrast, arginine.HCl exerts a peptide and cosolute concentration–dependent effect, confirming that protein stability in the presence of cosolute is strongly influenced by polypeptide chain length and charge (38).

The trends in the diffusion interaction parameter, *k*_*D*_, in the presence of cosolutes highlighted proline’s ability to decrease attractive intermolecular interactions between GLP-1am molecules and the propensity to grow into large aggregates. The mechanisms seem to involve stabilization of early soluble oligomeric species, given that (i) proline displays a general effect of reducing the measured *R*_h_ of GLP-1am; (ii) favors a dimeric state at low GLP-1am concentrations, and (iii) slows down the growth of aggregates. Low molecular weight species of GLP-1 analogs have been previously shown to inhibit peptide aggregation (14). Overall, the effect of proline on GLP-1am oligomerization agrees with its ability to inhibit protein aggregation *in vivo* and *in vitro* at early stages of aggregation but not when larger aggregates have formed (39).

The mechanism by which proline stabilizes GLP-1am early oligomers might involve one or multiple properties of this cosolute. We now know that in the glucagon family, the aggregation-prone regions include aromatic and hydrophobic amino acids (the xFxxWL hexapeptide), which drive the formation of reversible amyloids, the form in which these hormones are stored in acidic secretion vesicles (40). In general, proline is excluded from the protein backbone (41) and interacts preferentially with aromatic side chains based on the ability of the proline ring to form CH–π interactions (30, 42, 43). These properties make proline a key osmolyte for protein refolding (44, 45). Notably, proline has been shown to reduce protein–protein interactions between globular proteins (46, 47). Thus, we can speculate that proline might favor GLP-1am conformations that are less competent to aggregate, perhaps *via* excluded-volume effects (48). Last, proline can be effective in reducing the viscosity of proteins (49), but we exclude this effect as we do not observe any peptide viscosity changes with this cosolute (Fig. S9, *B*–*D*).

The pH dependence of proline stabilization has been less studied; thus, it is more difficult to explain. Previous observations on monoclonal antibody stability showed that proline, at the same concentration, can inhibit aggregation at pH 5 in histidine buffer (49) where antibody aggregation was less of an issue. Thus, the activity of proline may depend on the ability of proteins to form soluble low molecular weight oligomers with specific electrophoretic properties, like those conferred by the conformation adopted by GLP-1am at low concentrations and pH 5 (Table S5). This is supported by recent evidence that osmolytes can solubilize proteins only if they possess a certain degree of globularity (50).

Overall, we have shown that the cosolute effect and its trends are reflected by the reduction in the magnitude of the negative *k*_*D*_ with proline, but an increase in the magnitude of the negative *k*_*D*_ with NaCl and arginine.HCl, when compared with GLP-1am in buffer alone. The *k*_*D*_ encompasses electrostatic and hydrodynamic effects, which control protein diffusion (51). In the case of proline, its low ionic strength implies that diffusion of GLP-1am is driven less by electrostatics and more by protein–protein interactions. The presence of effective thresholds for cosolutes was previously observed also for glucagon (52) and likely reflects the dual ability of the guanidinium group of arginine.HCl and the proline ring (30) to engage with aromatic side chains as well as osmolyte effects.

In conclusion, we have shown that in conditions mimicking storage in secretory granules, GLP-1am exists as soluble discrete n-mers that assemble into nanosheets in quiescent conditions, at 25 °C (Fig. 5*G*). The addition of proline to GLP-1am solutions favors the lower oligomerization states and delays the growth into nanostructures. Conversely, ionic cosolutes, such as NaCl or arginine.HCl, promote fast aggregation of GLP-1am, indicating how screening of electrostatics promotes self-assembly. This work also shows that a combination of techniques is necessary to disentangle the complex behavior of protein self-association mechanisms in the presence of cosolutes and at peptide concentrations that are relevant to biology and pharmaceutical formulations. Last, we highlighted how the colloidal stability of a peptide can be measured by the interaction parameter, *k*_*D*_. This parameter might be used to quantify how effective a cosolute will be in reducing the aggregation of a given peptide.

## Experimental procedures

C-terminally amidated GLP-1am was purchased from two suppliers, GL Biochem Ltd (batch 1) and GenScript (batch 2). Both products were synthesized using solid-phase peptide synthesis, purified by reverse-phase HPLC and supplied as acetate salts. The GL Biochem batch had a reported purity of 95.29% as evaluated by reverse-phase HPLC. The company did not specify the residual content of counterions after purification. Two preparations were purchased from GenScript. Preparation 1 was 97.2% pure with a residual content of 0.45% TFA, 0.06% acetate, and 0.6% chloride. Preparation 2 was 95.1% pure with residual content of 0.62% TFA, 1.58% acetate, and 0.31% chloride. Quality control was performed by GenScript. No further purification on either batch was performed. Preparation 1 was used for experiments in Figures 3, 4, *E* and *F*, 5, S3, *E* and *F*, and S6 (except for SF6C). Preparation 2 was used for experiments in Figures S7*B*, C, *G*–*I*, and S8 (except for C and D).

### Buffer preparation

Sodium acetate buffer (25 mM sodium acetate, pH 5) was prepared from 8 mmol sodium acetate (≥99%, Sigma–Aldrich) and 4 mmol glacial acetic acid (≥99%, Thermo Fisher Scientific). The solution did not require further pH adjustment. Sodium phosphate buffer (25 mM, pH 8) was prepared from 0.0016 mol of sodium phosphate monobasic anhydrous (≥99%, Sigma–Aldrich) and 0.0233 mol of the basic component sodium phosphate dibasic heptahydrate (American Chemical Society grade, Sigma–Aldrich). The pH was adjusted using 12.5 M sodium hydroxide.

### Peptide stock preparation

GLP-1am was stored in lyophilized powder form at −20 °C, in tightly sealed and parafilmed vials, in a box containing silica gel. Stock solutions were prepared freshly on the day of measurement.

GL Biochem batch (batch 1): lyophilized GLP-1am was dissolved in 25 mM acetate buffer, pH 5, where it was soluble up to 12 mg mL^–1^. All solutions were prepared from this initial stock concentration to maintain consistency across datasets.

GenScript batch (batch 2): the lyophilized peptide from GenScript was soluble in 25 mM sodium acetate, pH 5, to a concentration of 4 mg mL^–1^ since opalescence was present above this concentration. Warming of the stock solution to 55 °C at a rate of 1 °C min^-1^ and holding for 1′ cleared the opalescence and increased solubility up to 12 mg mL^–1^.

Stock solutions were filtered through 0.2 μm polyethersulfone filters (Thomson) before measurements. The concentration was determined by measuring the absorbance at 280 nm of the stock solutions, using a calculated theoretical extinction coefficient of 6990 M^–1^ cm^–1^. The absorbance was measured after dilution using a NanoDrop One UV–Vis Spectrophotometer (ThermoFisher Scientific) or a VersaWave UV/Vis spectrophotometer.

### Cosolute stock preparation

Stocks of l-arginine.HCl, l-proline, and NaCl (Sigma–Aldrich) were made by dissolving the respective cosolute in the appropriate buffer to give a 600 mM concentration and subsequently filtered through 0.2 μm polyethersulfone filters (Millex).

### DLS and nanoDSF

Individual samples were prepared by dilution of peptide stock or cosolute stock in sodium acetate buffer to give the desired peptide or cosolute concentration. High-throughput combined DLS/nanoDSF measurements were performed using a Prometheus Panta (NanoTemper Technologies), which uses a 405 nm laser with a 147° scattering angle. Approximately 10 μl of sample was loaded into NanoTemper standard capillaries, and DLS was performed at 20 °C with 10 acquisitions, each with an acquisition time of 5 s. Thermal stability was measured by nanoDSF, monitoring the intrinsic fluorescence at emission wavelengths of 330 and 350 nm (excitation at 280 nm) *via* a thermal ramp of 1 °C min^–1^ from 20 to 95 °C. The diffusion interaction parameter *k*_*D*_ was determined using a concentration series of five different peptide concentrations between 1 and 8 mg mL^-1^, with 10 acquisitions of 5 s acquisition time. All formulations were measured in independent triplicate. The onset of aggregation was measured by monitoring turbidity and cumulant radius simultaneously. Data analysis was performed using PR.Analysis software, version 1.7.1 (NanoTemper Technologies). Selected samples were measured again in low-throughput DLS using a Mobius (Wyatt Technology) with a 532 nm laser at 163.5° scattering angle, more sensitive to polydisperse samples. For all samples, the dispersant viscosity (including cosolutes when appropriate) was used to calculate the hydrodynamic radii of the samples.

### Diffusion interaction parameter (*k*_*D*_) determination

*K*_*D*_ calculations were performed using the diffusion coefficient of the major species (peak 1, *R*_h_ = 2.1 nm) from the hydrodynamic radius using the Stokes–Einstein Equation:

$$D_{c}=\frac{k_{B}T}{6\pi \eta R_{h}}$$

where *D*_*c*_ is the translational diffusion coefficient, *k*_*B*_ is the Boltzmann constant, *T* is the temperature at which the DLS was taken, η is the viscosity of the buffer, and *R*_h_ is the hydrodynamic radius. The subsequent diffusion coefficient was utilized in linear regression.

The diffusion interaction parameter was determined by linear regression of the diffusion coefficients of solutions with varying peptide concentrations, with or without cosolutes. The equation used to fit the data is:

$$D=D_{0}\left(1+k_{D}c\right)$$

where *D* is the diffusion coefficient of the peptide in solution, *D*_0_ is the diffusion coefficient at infinite dilution, and *c* is the concentration of the protein in solution.

A total of five capillaries per concentration (except for 6 mg mL^-1^) were repeated over 2 days. Acquisitions with autocorrelation curves giving nonrandom residuals were excluded from the analysis. For each capillary, the average of 10 acquisitions was displayed as a data point. Data fitting was performed using the linear regression function from the SciPy (version 1.11.4) package in Python (version 3.11.7). The standard error was calculated by error propagation using the errors from the linear regression of the slope and the intercept.

### Zeta-potential determination

Zeta-potential determination was performed at 25 °C using a Mobius (Wyatt Technology). All data analyses were performed using Dynamics Software, version 8.1.2.144 (Wyatt Technology). The zeta potential was measured at a peptide concentration of 0.3 mg/ml in pH ranges from 3.75 to 5 (sodium acetate, 25 mM) and 6.2 to 8 (sodium phosphate, 25 mM). The electric field frequency was 10.0 Hz, and the voltage amplitude was 2.5 V.

### Dispersant viscosity measurement

The viscosity of the dispersants was measured at 20 °C using the Prometheus Panta (NanoTemper Technologies) and 100 nm polystyrene 3000 Series Nanosphere Size Standards (Thermo Scientific). Nanospheres were diluted 1:1000 into samples containing cosolute and buffer (Table S6). A buffer of known viscosity was also used as a reference; in this case, it was either sodium acetate (25 mM) or phosphate-buffered saline. The size of the nanospheres was compared, and the following equation was used to estimate the viscosity:

$$\text{Viscosity}=\text{viscosit}y_{\text{reference}}\times \frac{R_{h}}{R_{h\;\left(\text{reference}\right)}}$$

A Honeybun rheometer (Unchained Labs) was used to confirm the reliability of some of the values measured with the nanospheres. Data analysis was performed using the Honeybun software (Unchained Labs).

### Kinetics of aggregation by turbidity

Kinetics of aggregation was monitored by measuring the turbidity of samples by UV spectroscopy, either using a Cary 3500 Multicell UV–Vis Spectrophotometer (Agilent Technologies) or a FLUOstar Omega plate reader (BMG LabTech). The absorbance at 350 nm was monitored every 5 min, without agitation, at 25 °C.

### Kinetics of amyloid formation by thioflavin T binding and low-throughput intrinsic tryptophan fluorescence

Thioflavin (ThT) binding was monitored using a Fluoromax 4 (Horiba). GLP-1am at 1 mg mL^-1^ was incubated with 100 μM ThT, at 37 °C in a shaking incubator with 180 rpm. Detection of ThT binding was performed by recording the fluorescence emission at 482 nm with an excitation wavelength of 448 nm. Fluorescence was measured with a 5 nm response.

Samples containing 2 mg mL^-1^ were incubated at 25 °C in 25 mM sodium acetate, pH 5, for 4 days. Fluorescence was recorded each day using a Fluoromax 4 (Horiba). Changes in intrinsic Trp fluorescence were recorded by measuring emission at 300 to 400 nm with excitation at 280 nm with a 2 nm bandwidth.

### SEC by FPLC

Analytical SEC was performed using an ÄKTA Pure 25 (Cytiva) using a Superdex 75 10/300 GL (Cytiva) with a flow rate of 0.7 ml/min, with the mobile phase having the same composition as the formulation buffer. The Gel Filtration Calibration Kit (Low-Weight Molecular; Cytiva) containing aprotinin, ribonuclease, carbonic anhydrase, and ovalbumin was run under the same elution conditions as the experiments. The molecular weight plotted as a function of elution volume (logM=b−cV) was used to estimate the size of the GLP-1am species present (Fig. S4*F*).

### Asymmetric flow field flow fractionation

AF4–MALS was performed using an Infinity II HPLC (Agilent Technologies) and a 2 kDa regenerated cellulose membrane in a variable height, short channel with a 275 μm spacer. Samples were introduced using a 1260 Vialsampler and monitored with the following in-line detectors: 1260 DAD UV–Vis spectrophotometer, 1260 fluorescence, DAWN (Wyatt Technology), and Optilab (Wyatt Technology). Channel flow rates were controlled by an Eclipse (Wyatt Technology). The AF4 method used is in Table S7 with a focusing position of 25%.

### Circular dichroism

CD spectra were measured on a Jasco J-810 CD spectrometer (Jasco). Far-UV CD spectra were measured in a 1-mm pathlength cuvette with 1-nm step size and with a 1-nm spectral bandwidth and a monomer concentration of 0.3 mg mL^-1^. Near-UV CD spectra were measured with 1-nm step size and 1-nm spectral bandwidth in a 2-mm pathlength cuvette and a monomer concentration of 2 mg mL^-1^. The recording was performed in step mode with 8 s response time. All measurements were performed at 20 °C.

The millidegree units were converted into molar ellipticity after buffer subtraction using

$$\theta =\frac{mdeg}{10*N*c*l}$$

where N is the number of peptide bonds, l is the pathlength in cm, and *c* is the concentration of peptide monomer in mol L^-1^.

### Analytical ultracentrifugation

Sedimentation velocity analytical ultracentrifugation was performed on a Beckman Optima analytical ultracentrifuge using absorbance optics set at 290 nm. Samples were loaded into two-sector cells with quartz glass windows, and the samples were centrifuged at 50,000 RPM using an 8-hole An50Ti rotor. Scans were set to be every 60 s for a total of 400 scans.

Analysis was performed using Sedfit (53). Sedimentation coefficients were corrected for buffer composition to standard conditions.

### Negative staining and transmission electron microscopy

Negative staining was performed using freshly glow-discharged, carbon-coated copper grids. The sample was applied for 1.5 min and blotted away. Grids were washed twice with water, followed by two washes with 0.75% uranyl formate, with the second staining step in 0.75% uranyl formate for 20 s.

Transmission electron microscopy studies were conducted using a JEOL JEM-120i electron microscope operating at 80 kV and equipped with a SightSKY (EM-04500SKY) 19 megapixel camera.

## Data availability

Data are available to be shared upon request. Please contact b.ciani@sheffield.ac.uk.

## Supporting information

This article contains supporting information (54).

## Conflict of interest

R. M., R. K. S., and V. L. are employees of AstraZeneca. N. B. P. A. is an employee of NanoTemper Technologies GmbH. All other authors declare that they have no conflicts of interest with the contents of this article.
