# Supplementary material for: Cosolute effects reveal the nature of weak forces governing GLP-1 oligomer stability
Source: J Biol Chem. 2026 Feb 2;302(4):111223. doi: 10.1016/j.jbc.2026.111223 (PMC13010907; doi:10.1016/j.jbc.2026.111223)
Supplement: Supplementary Material [file mmc1.pdf]

Supporting Information

Co-solute Effects Reveal the Nature of Weak Forces Governing GLP-1 Oligomers Stability

Anyah Settle[1], Rahul Mishra[2], Ramesh-Kumar Shanmugam[3], Viv Lindo[2], Nathan B P Adams[4], Thomas A Jowitt[5], Tuck Seng Wong[6*] and Barbara Ciani[1*].

1 Centre for Chemical Biology, School of Mathematical and Physical Science, University of Sheffield, Sheffield S3 7HF, UK

2. BioPharmaceuticals Development, Analytical Sciences, AstraZeneca, Cambridge CB2 0AA, United Kingdom, UK

3. Biopharmaceutical Development, Dosage Form Design and Development, AstraZeneca, Cambridge CB2 0AA, UK

4. NanoTemper Technologies GmbH, Tölzer Str 1, 81379, Munich, Germany

5. Wellcome Centre for Cell-Matrix Research, Faculty of Biology, Medicine and Health, Manchester Academic Health Science Centre, University of Manchester, Manchester M13 9PT, UK

6. School of Chemical, Materials and Biological Engineering, University of Sheffield, Sir Robert Hadfield Building, Mappin Street, Sheffield, S1 3JD, UK

Table S1. R_h of GLP-1am main species by DLS (Mobius, Wyatt).

[GLP-1am (pH 5)] / mg mL^{-1}	R_h	% mass	Pd (%)
1	2.6 ± 0.1	99.9	18
2	2.9 ± 0.2	99.9	22
4	3.1 ± 0.3	99.9	19
6	3.3 ± 0.6	99.9	22
8	3.4 ± 0.6	99.9	18
[GLP-1am (pH 8)] / mg mL^{-1}	R_h	% mass	Pd (%)
1	2.7	100	15
2	3.1 ± 0.1	100	23
4	3.4 ± 0.1	100	20
6	3.7 ± 1.1	79	20
8	3.9 ± 1.6	86	20

Table S2. Asymmetric flow field-flow fractionation analysis of GLP-1am. The oligomeric state was calculated based on the MW GLP-1am monomer being 3354.68 Da.

**[GLP-1am] / mg mL^{-1}**	**MW / kDa**	**Uncertainty**	**Pd**	**N-mer**
1 (pH 5)	6.3	17.60%	1.065	1.8
2 (pH 5)	8.2	17.30%	1.086	2.4
4 (pH 5)	4.6	16.20%	1.017	1.4
2 (pH 8)	37.8	34.50%	1.613	11.2

Table S3. Sedimentation velocity analytical ultracentrifugation analysis of GLP-1am in 25 mM acetate buffer pH 5. The oligomer number (in brackets) was calculated based on the MW GLP-1am monomer being 3354.68 Da.

[GLP-1am] / mg mL^{-1}		Peak 1	Peak 2	Peak 3	Peak 4
2	$s_{20,w}$ / S	0.1	1.4	2.0	
	MW / kDa	NA	13 (3.9)	24 (7.2)	
4	$s_{20,w}$ / S	0.1	1.19	2.03	2.7
	MW / kDa	NA	13.7 (4.1)	29.1 (8.7)	46.5 (13.9)
2 (100 mM proline)	$s_{20,w}$ / S	0.1	1.46	2.03	
	MW / kDa	NA	13.5 (4)	21 (6.3)	
4 (18 hrs, 25 °C)	$s_{20,w}$ /S	0.1	1.16	2.02	
	MW / kDa	NA	12.1 (3.6)	27.2 (8.1)	
4 (100 mM proline)	$s_{20,w}$ /S	0.1	1.2	2.1	
	MW / kDa	NA	13.5 (4)	30.6 (9.1)	
4 (100 mM proline, 18 hrs, 25 °C)	$s_{20,w}$ /S	0.1	1.15	1.91	
	MW / kDa	NA	12 (3.6)	25.5 (7.6)	

Table S4. Size-exclusion chromatography analysis of GLP-1am. The oligomeric state (in brackets) was calculated based on the MW GLP-1am monomer being 3354.68 Da.

[GLP-1am] / mg mL^{-1}	MW / Da	n-mer
0.3 (pH 5)	5970	1.8
0.3 (pH 5, 100 mM proline)	8026	2.4
0.3 (pH 8)	4660	1.4
2 (pH 5)	8770	2.6
2 (pH 5, 100 mM proline)*	7015	2.1

* Mobile phase: 25 mM acetate buffer pH 5, 100 mM proline

Table S5. ζ-Potential of GLP-1am in 25 mM acetate buffer pH 5, at 20 °C. One repeat only.

[GLP-1am] / mg mL^{-1}	ζ-Potential/ mV	Main peak R_h / nm	%Pd	%Mass
1	0.3	2.5	15.8	99.6
2	9.48	2.8	15.5	99.9
4	0.53	3.2	24.8	100
8	2.61	3.6	23.5	100

Table S6. Estimated viscosity of solutions in 25 mM acetate pH 5 (cP) using nanospheres compared to rheometer.

[co-solute] / mM	NaCl	Arginine.HCl	Pro
12.5	1.012 ± 0.03		1.008 ± 0.03
25	1.012 ± 0.03	1.03 ±0.03 (1.041 ±0.004)*	1.016 ± 0.03
50	1.018 ± 0.05	1.06 ±0.03 (1.084 ±0.005)*	1.013 ± 0.03
100	1.007± 0.03	1.06 ±0.03 (1.067 ±0.002)*	1.038 ± 0.03
150	1.013 ± 0.03	1.07 ±0.03 (1.084 ±0.006)*	1.048 ± 0.03
200		1.2 ±0.03 (1.07 ±0.02)*	

* Data from Honeybun at 20 °C

Table S7. AF4 method for separation of GLP-1am in 25 mM acetate buffer, pH 5.

Mode	Duration / min	Cross Flow Start / mL min^{-1}	Cross Flow Stop / mL min^{-1}	Flow Profile
**Elution**	1	0	3	Linear
**Elution**	2	3	3	Constant
**Focus**	1	3	3	Constant
**Focus Inject**	3	3	3	Constant
**Focus**	2	3	3	Constant
**Elution**	15	3	3	Constant
**Elution**	1	3	0	Linear
**Elution**	5	0	0	Constant
**Elution Inject**	5	0	0	Constant

Figure S1.

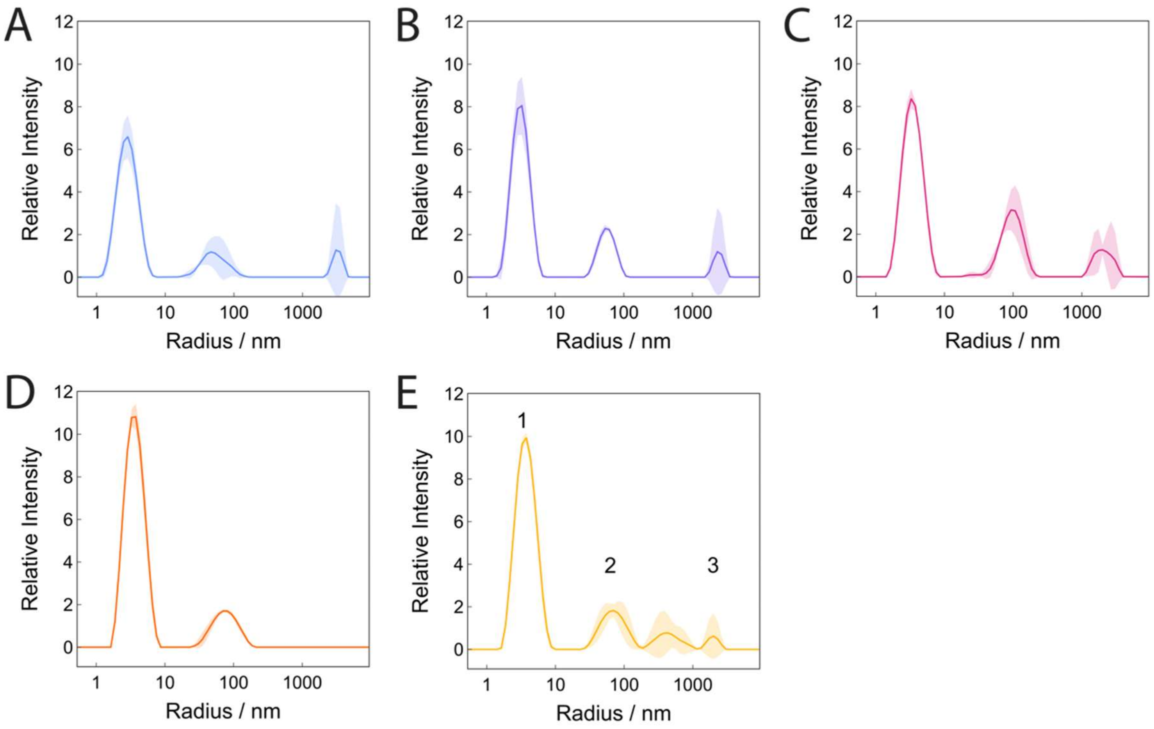

Figure S1. Size distribution by relative intensity of scattered light of GLP-1am samples in 25 mM acetate buffer pH 5 and 20 °C at (A) 1, (B) 2, (C) 4, (D) 6 and (E) 8 mg mL^{-1}. The standard deviation of 3 repeats is shown as shaded regions. Measurements taken with the Prometheus Panta (NanoTemper).

Figure S2

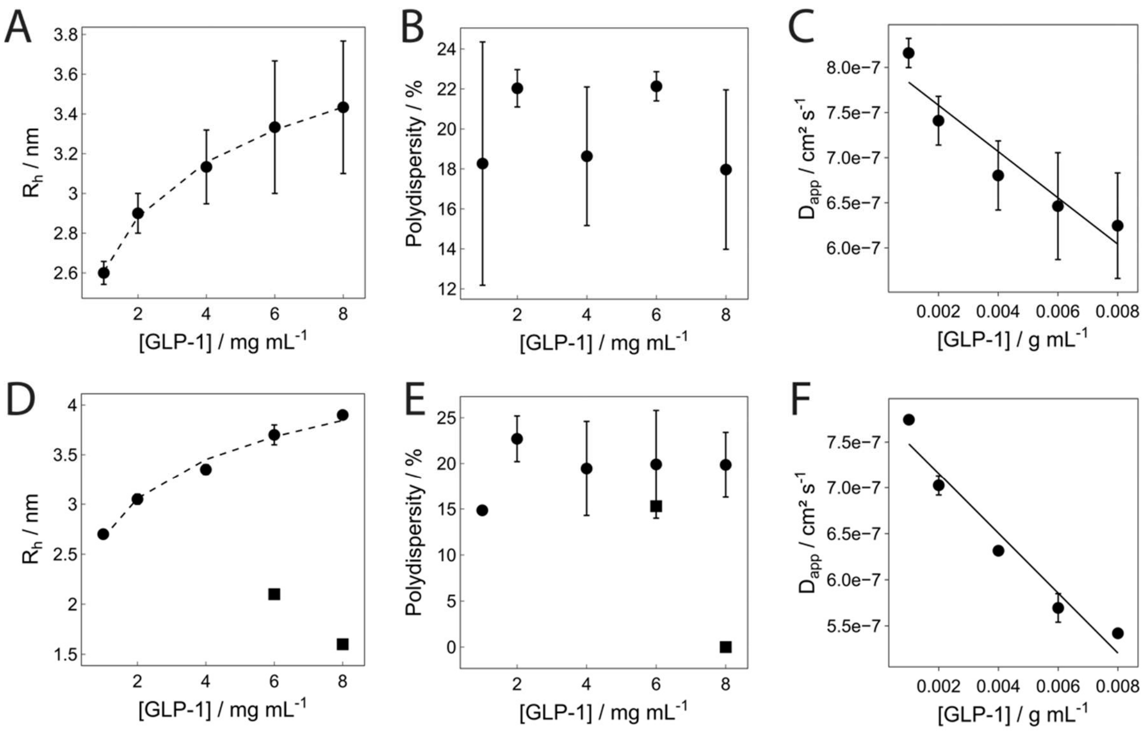

Figure S2. (A) Apparent hydrodynamic radius, (B) polydispersity and (C) diffusion coefficient for GLP-1am in 25 mM acetate buffer pH 5 and 20 °C as function of its concentration used to calculate the diffusion interaction parameter, k_D ($k_D = -32 \pm 9$ mL g^{-1}). (D) Apparent hydrodynamic radius and (E) polydispersity and (F) diffusion coefficient of GLP-1am in 25 mM phosphate buffer pH 8 and 20 °C as function of its concentration used to calculate the diffusion interaction parameter, k_D ($k_D = -42 \pm 8$ mL g^{-1}). Samples at 6 and 8 mg mL^{-1} show GLP-1am as a main specie (filled circles; 70% mass) and minor specie (filled squares; 30% mass). Measurements taken with Mobius (Wyatt) using a 532 nm laser with a 163.5° scattering angle. Average and standard deviation of 3 technical repeats shown.

Figure S3

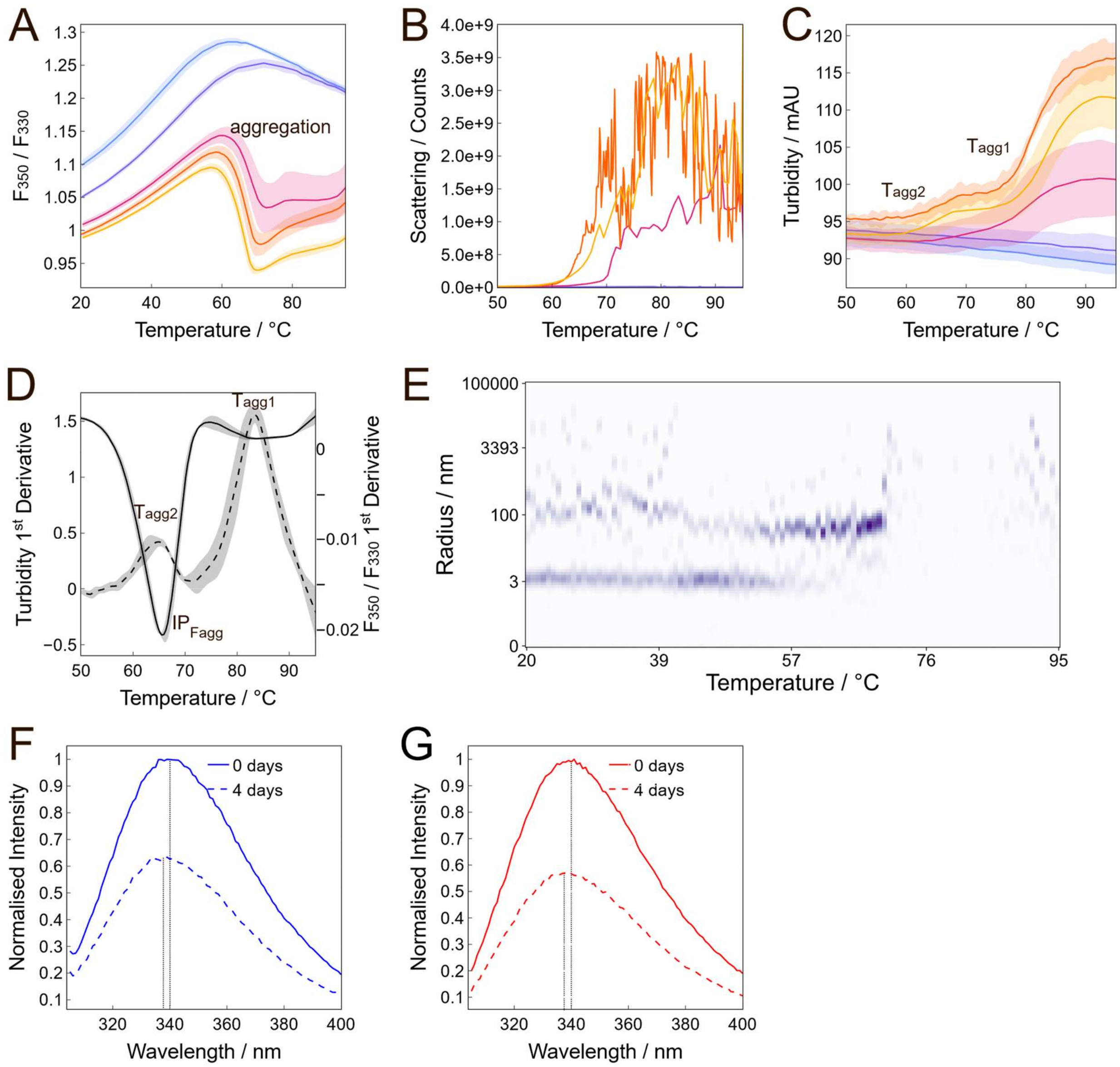

Figure S3. (A) Ratio of 350 nm to 330 nm of the intrinsic fluorescence vs temperature for GLP-1am at 1 mg mL^{-1} (blue), 2 mg mL^{-1} (purple), 4 mg mL^{-1} (pink), 6 mg mL^{-1} (orange) and 8 mg mL^{-1} (yellow) in 25 mM acetate buffer pH 5. (B) Scattering as a function of peptide concentration for GLP-1am at 4 mg mL^{-1} (pink), 6 mg mL^{-1} (orange) and 8 mg mL^{-1} (yellow) in 25 mM acetate buffer pH 5. (C) Turbidity as function of peptide concentration for GLP-1am at 1 mg mL^{-1} (blue), 2 mg mL^{-1} (purple), 4 mg mL^{-1} (pink), 6 mg mL^{-1} (orange) and 8 mg mL^{-1} (yellow) in 25 mM acetate buffer pH 5. (D) Correlation between the first derivative of the intrinsic fluorescence 330/350 ratio (solid line) with the first derivative of turbidity (dashed line) for 4 mg mL^{-1} GLP-1am in 25 mM acetate buffer pH 5 and 20 °C. Average and standard deviation of 3 repeats shown. (E) Size distribution from DLS vs temperature for GLP-1am at 4 mg mL^{-1}. (F) Intrinsic tryptophan fluorescence of 2 mg mL^{-1} GLP-1am, after 4 days incubation in 25 mM acetate buffer pH 5, in quiescent conditions, at 25 °C. Emission intensity normalised to the highest signal. (G) Intrinsic tryptophan fluorescence of 2 mg mL^{-1} GLP-1am, after 4 days incubation in 25 mM phosphate buffer pH 8, in quiescent conditions, at 25 °C. Emission intensity normalised to the highest signal.

Figure S4

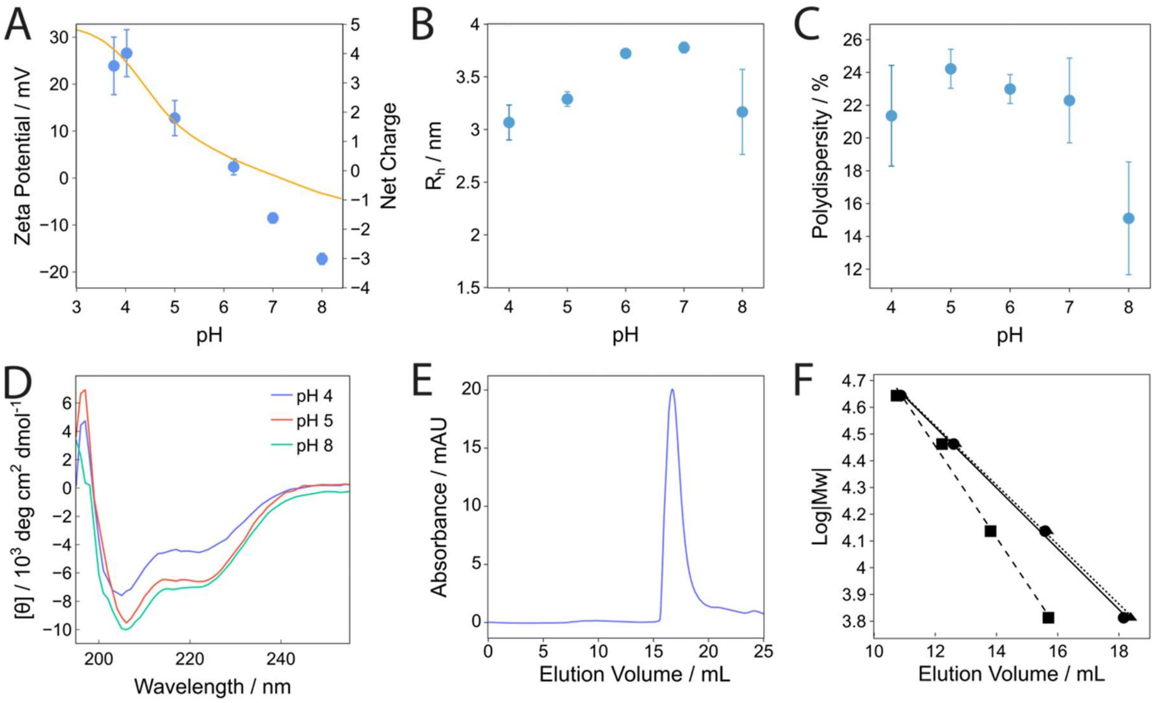

Figure S4. (A) Calculated isoelectric point vs pH for GLP-1am overlaid to the measure ζ-Potential for a 0.3 mg mL^{-1} sample at 20 °C. Average and standard deviation of 3 repeats shown. Calculated pI = 6.765 (Expasy pKa data). Net charge = - 0.779 (pH 8); +1.195 (pH 5). Calculations performed with https://www.protpi.ch/Calculator/ProteinTool. (B) Apparent hydrodynamic radius and (C) polydispersity index of 2 mg mL^{-1} GLP-1am in 25 mM ammonium acetate buffer at 20 °C as function of pH. Measurements taken with Mobius (Wyatt). Average and standard deviation of 3 technical repeats shown. (D) pH-dependence of secondary structure content by circular dichroism of 0.3 mg mL^{-1} GLP-1am at 25 °C. (E) SEC of a 0.3 mg mL^{-1} GLP-1am fresh sample in 25 mM phosphate pH 8. (F) Calibration with MW standards of the Superdex 75 10/300 GL in 25 mM acetate buffer pH 5 (circles, solid line), 25 mM acetate buffer pH 5 with 100 mM proline (triangles, dotted line) and 25 mM phosphate buffer pH 8 (squares, dashed line). Aprotinin (6.5kDa), Ribonuclease A (13,7kDa), Carbonic Anhydrase (29kDa), Ovalalbumin (43kDa).

Figure S5

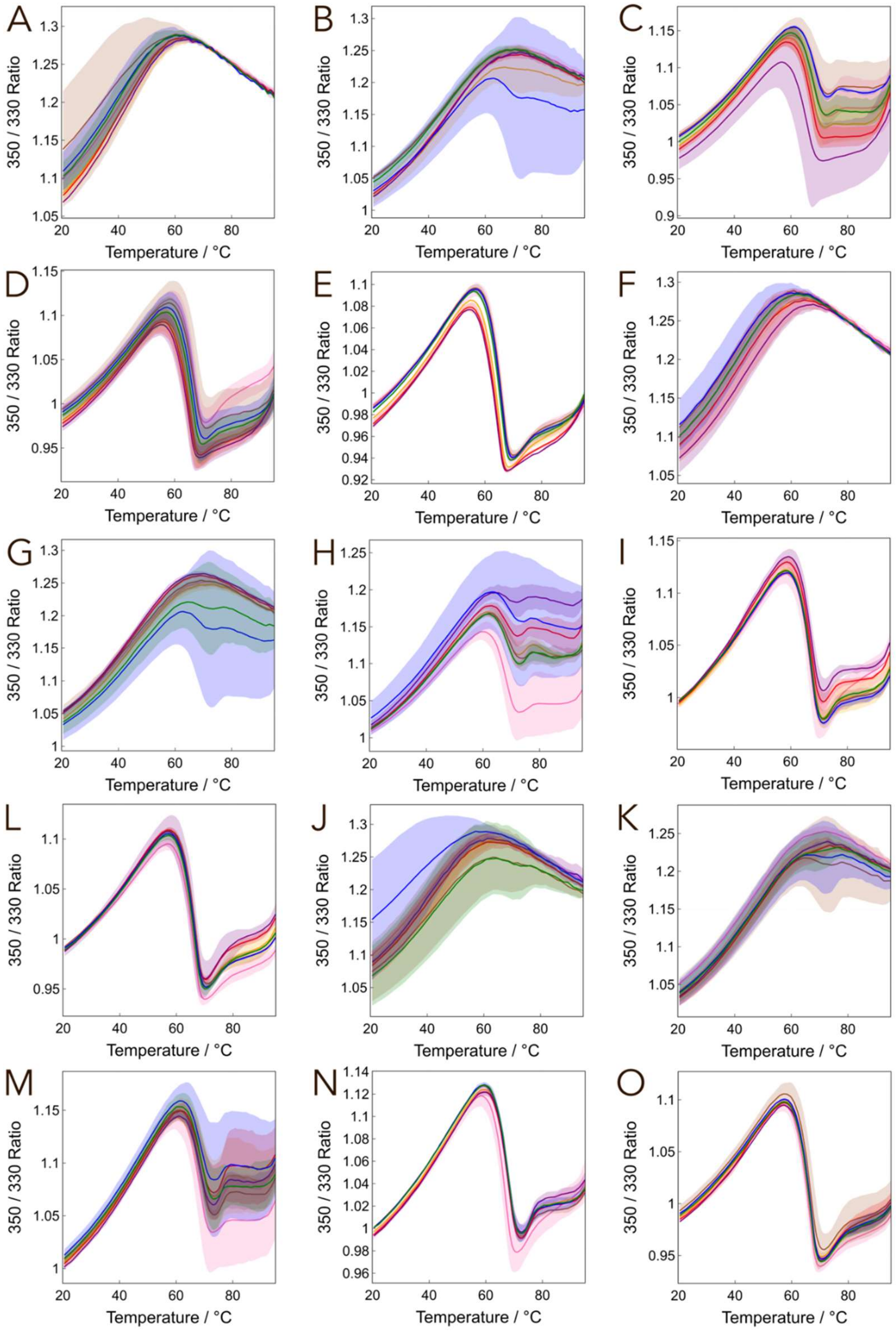

Figure S5. Thermal stability of GLP-1am in the presence of co-solutes measured by differential scanning fluorimetry (Prometheus Panta). (A-E) 1, 2, 4, 6, 8 mg mL^{-1} GLP-1am with sodium chloride. (F-L) 1, 2, 4, 6, 8 mg mL^{-1} GLP-1am with arginine.HCl. (M-Q) 1, 2, 4, 6, 8 mg mL^{-1} GLP-1am with proline. Excipient concentration: 12.5 mM (brown), 25 mM (blue), 50 mM (green), 100 mM (orange), 150 mM (red), 200 mM (purple), 25 mM acetate buffer pH 5 (pink).

Figure S6

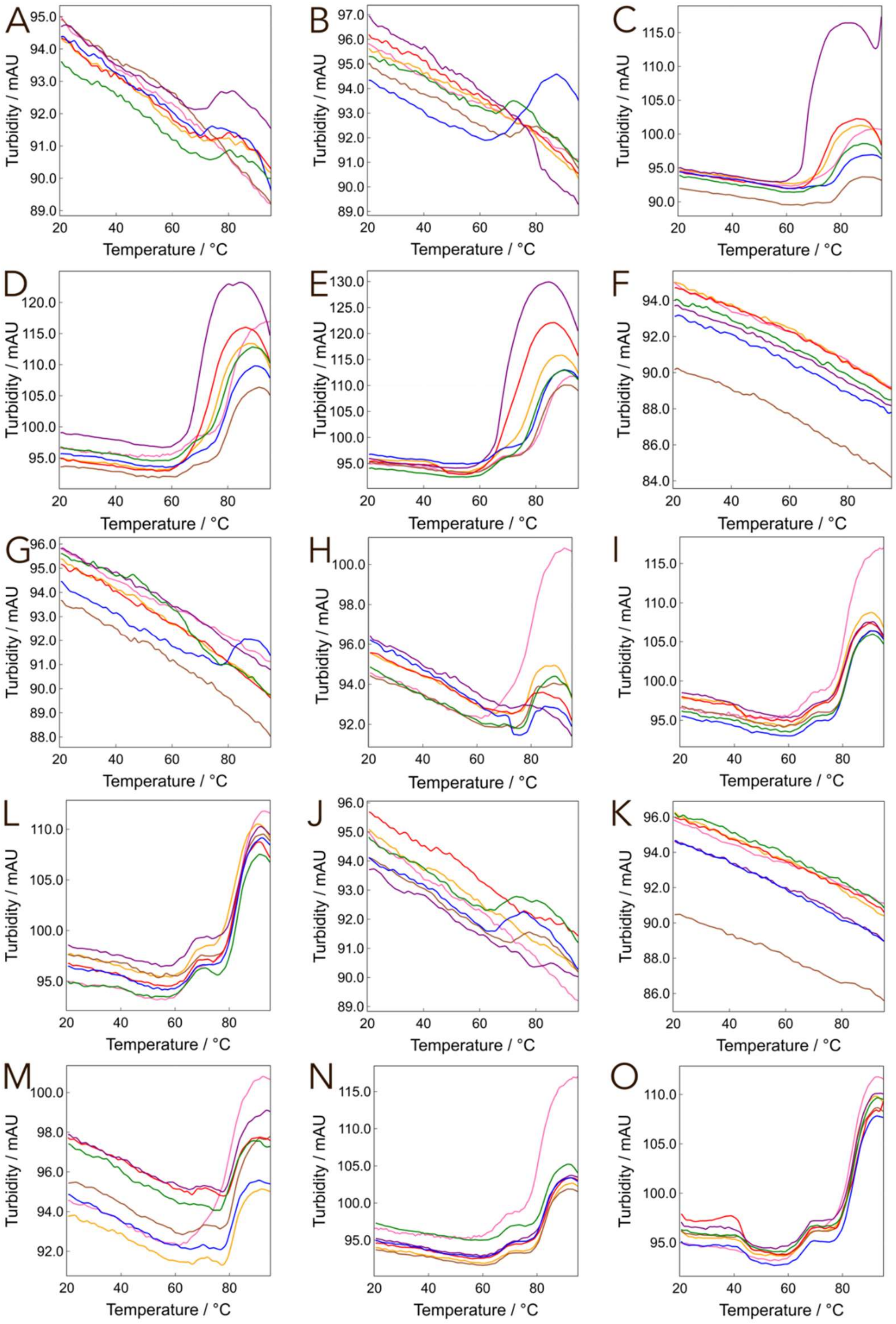

Figure S6. Turbidity of GLP-1am in the presence of co-solutes measured by backscattering (Prometheus Panta). (A-E) 1, 2, 4, 6, 8 mg mL^{-1} GLP-1am with sodium chloride. (F-L) 1, 2, 4, 6, 8 mg mL^{-1} GLP-1am with arginine.HCl. (M-Q) 1, 2, 4, 6, 8 mg mL^{-1} GLP-1am with proline. Excipient concentration: 12.5 mM (brown), 25 mM (blue), 50 mM (green), 100 mM (orange), 150 mM (red), 200 mM (purple), 25 mM acetate buffer pH 5 (pink).

Figure S7

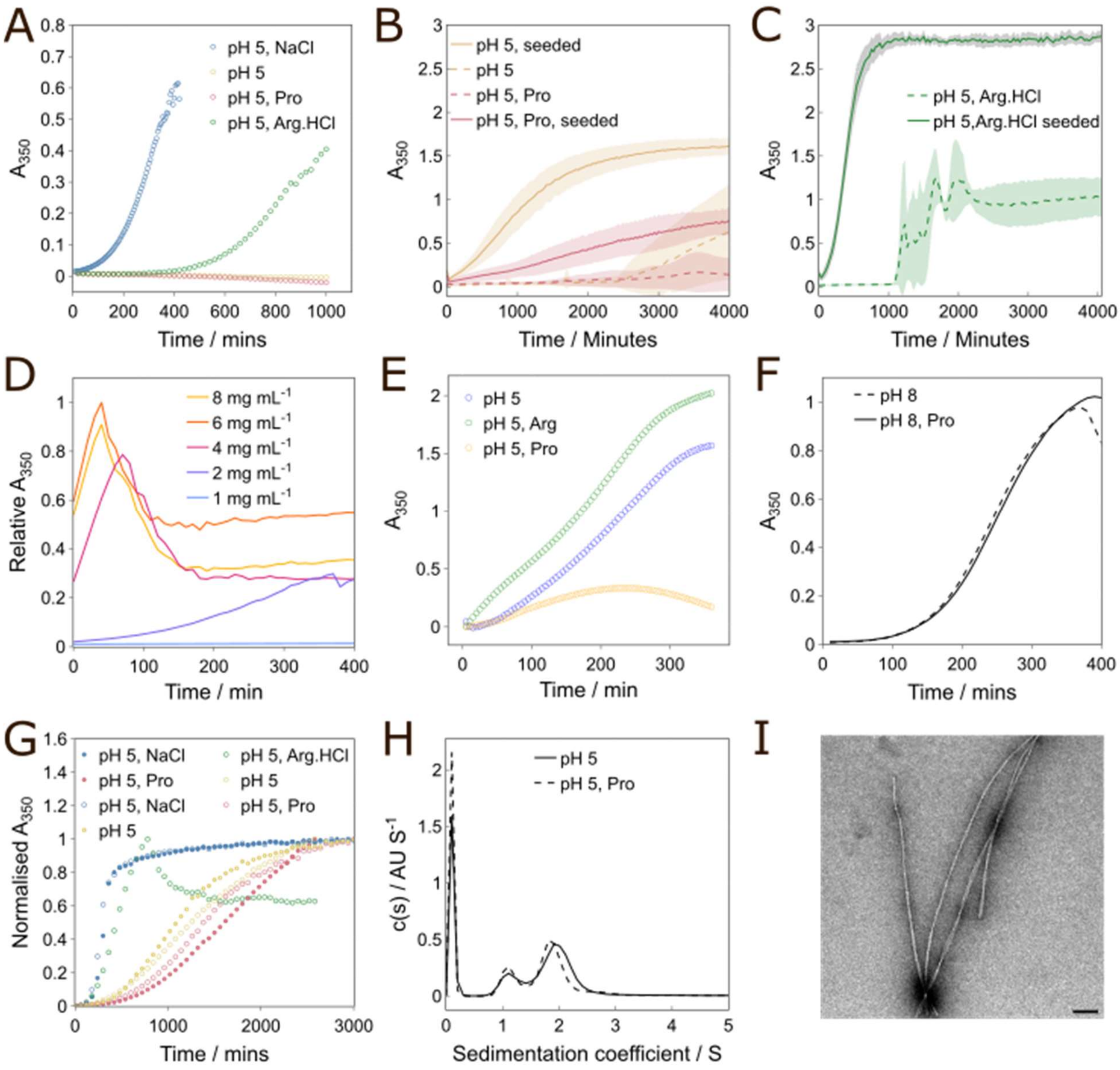

Figure S7. Aggregation kinetics at 25°C, monitored by turbidity with apparent UV absorbance at 350 nm. (A) 2 mg mL^{-1} GLP-1am (batch 1) in 25 mM acetate buffer pH 5 without and with co-solutes. (B) 4 mg mL^{-1} GLP-1am (batch 2) in 25 mM acetate buffer pH 5 without (red) and with (yellow) proline, with (solid lines) and without (dashed lines) 1% (v/v) of seeds. (C) 4 mg mL^{-1} GLP-1am (batch 2) with arginine amino acid with (solid line) and without (dashed line) 1% (v/v) of seeds. (D) Concentration-dependence of GLP-1am (batch 1) assembly in 25 mM phosphate buffer pH 8. (E) 6 mg mL^{-1} GLP-1am (batch 1) in 25 mM acetate buffer pH 5 with zwitterionic arginine and 100 mM proline. (F) 2 mg mL^{-1} GLP-1am (batch 1) in 25 mM phosphate buffer pH 8 with and without 100 mM proline. (G) Aggregation kinetics at 25 C of 2 mg mL^{-1} GLP-1am (batch 2) in 25 mM acetate buffer pH 5 with and without co-solutes. Absorbance at 350 nm was normalized to the highest and lowest value within each dataset. Each dataset (NaCl, proline, and acetate alone) was collected on the same day. Two datasets collected on different days were compared (solid and open circles). A single repeat for Arg.HCl is shown. (H) Sedimentation velocity of GLP-1am (batch 2) 4 mg mL^{-1} aggregated over 18 hours without and with 100 mM proline, in 25 mM acetate buffer at pH 5. (I) TEM of GLP-1am 1 mg mL^{-1} after 6 days incubation, at 25 °C, with agitation.

Figure S8

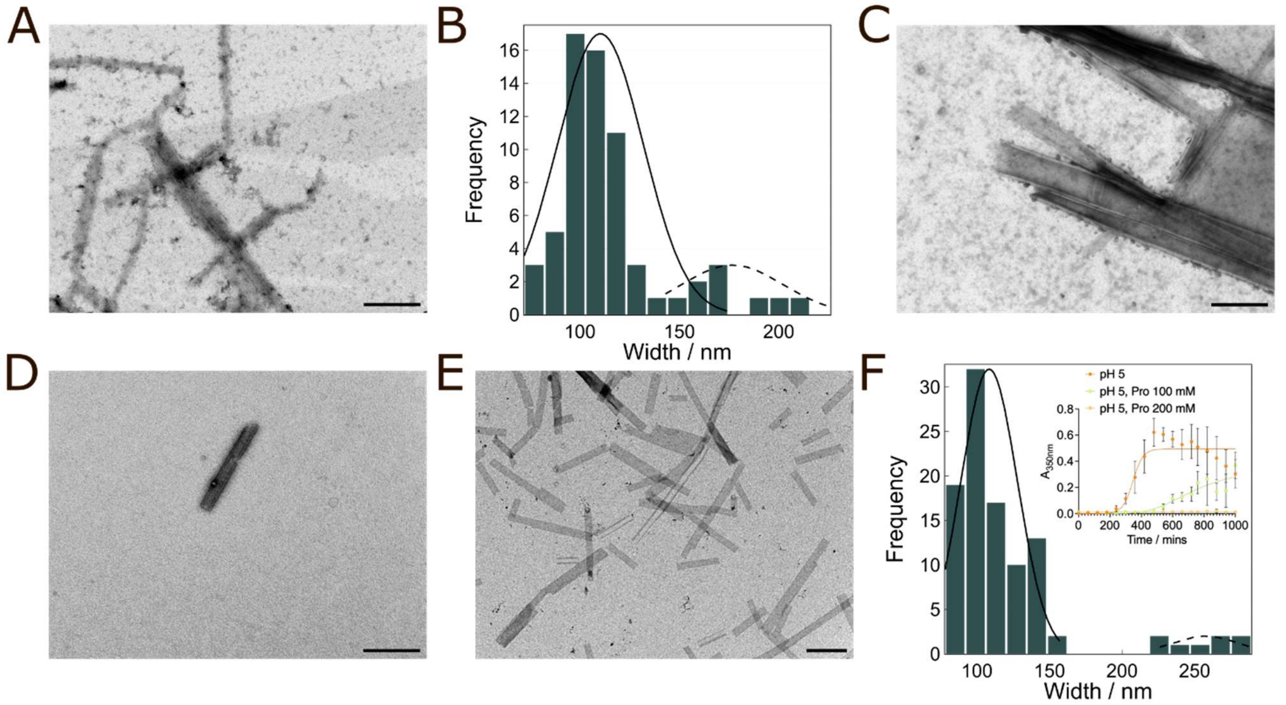

Figure S8. Transmission electron microscopy of GLP-1am incubated at 25 °C, without agitation. (A) 2 mg mL^{-1} in 25 mM phosphate buffer pH 8 for 18 hours (batch 2). (B) Width binned distribution of GLP-1am nanosheets of 2 mg mL^{-1} in 25 mM phosphate buffer pH 8 for 18 hours (batch 2). (C) 8 mg mL^{-1} GLP-1am matured at room temperature at pH 8, for 2 weeks (batch 1). (D) 8 mg mL^{-1} GLP-1 25 mM acetate buffer, pH 5 for 4 weeks (batch 1). Scale bar 500 nm. (E) Transmission electron microscopy of 2 mg mL^{-1} GLP-1am incubated in 25 mM sodium acetate pH 5 and 200 mM proline, for 18 hours (batch 2). Scale bar 500 nm. (F) Width binned distribution of GLP-1am nanosheets of 2 mg mL^{-1} in 25 mM sodium acetate buffer pH 5 with 200 mM proline, incubated for 18 hours (batch 2). The inset shows the impact of proline concentrations on the aggregation kinetics of 4 mg mL^{-1} GLP-1am at 25 °C, without agitation (batch 2).

Figure S9

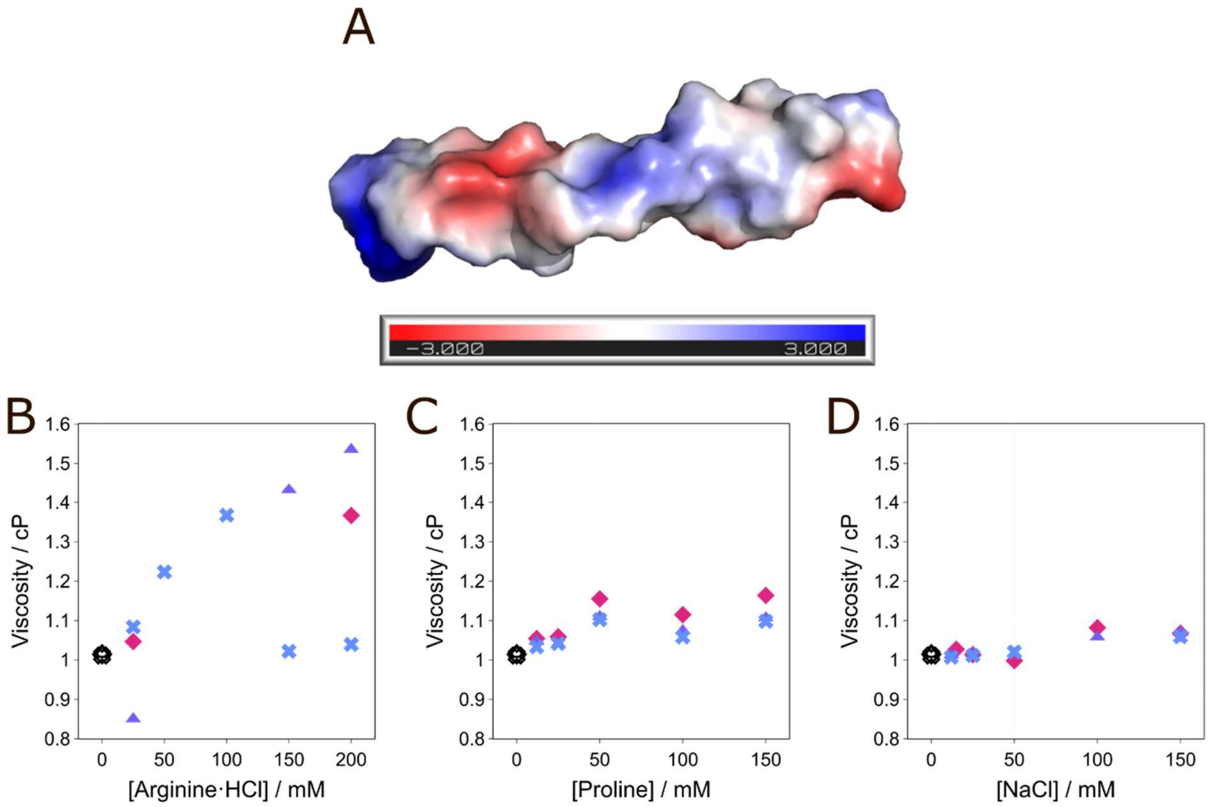

Figure S9. (A) Electrostatic surface of GLP-1am generated with APBS and depicted with Pymol. The model of GLP-1 7-37 amide was created with AlphaFold3 and the C-term amidation was introduced with UCSF Chimera. (B-D) Viscosity of GLP-1am samples vs arginine.HCl (B), proline (C) and (D) NaCl concentrations (see methods). The average of three technical repeats shown. GLP-1am 4 mg mL^{-1} (diamond, pink), 2 mg mL^{-1} (triangle, purple), 1 mg mL^{-1} (cross, blue). Compared with GLP-1am in acetate alone shown in black.
